# Supplementary material for: NT-proBNP Concentrations in the Umbilical Cord and Serum of Term Neonates: A Systematic Review and Meta-Analysis
Source: Diagnostics (Basel). 2022 Jun 8;12(6):1416. doi: 10.3390/diagnostics12061416 (PMC9222102; doi:10.3390/diagnostics12061416)
Supplement: Supplementary file 1 [file diagnostics-12-01416-s001.zip › diagnostics-1739527-supplementary.pdf]

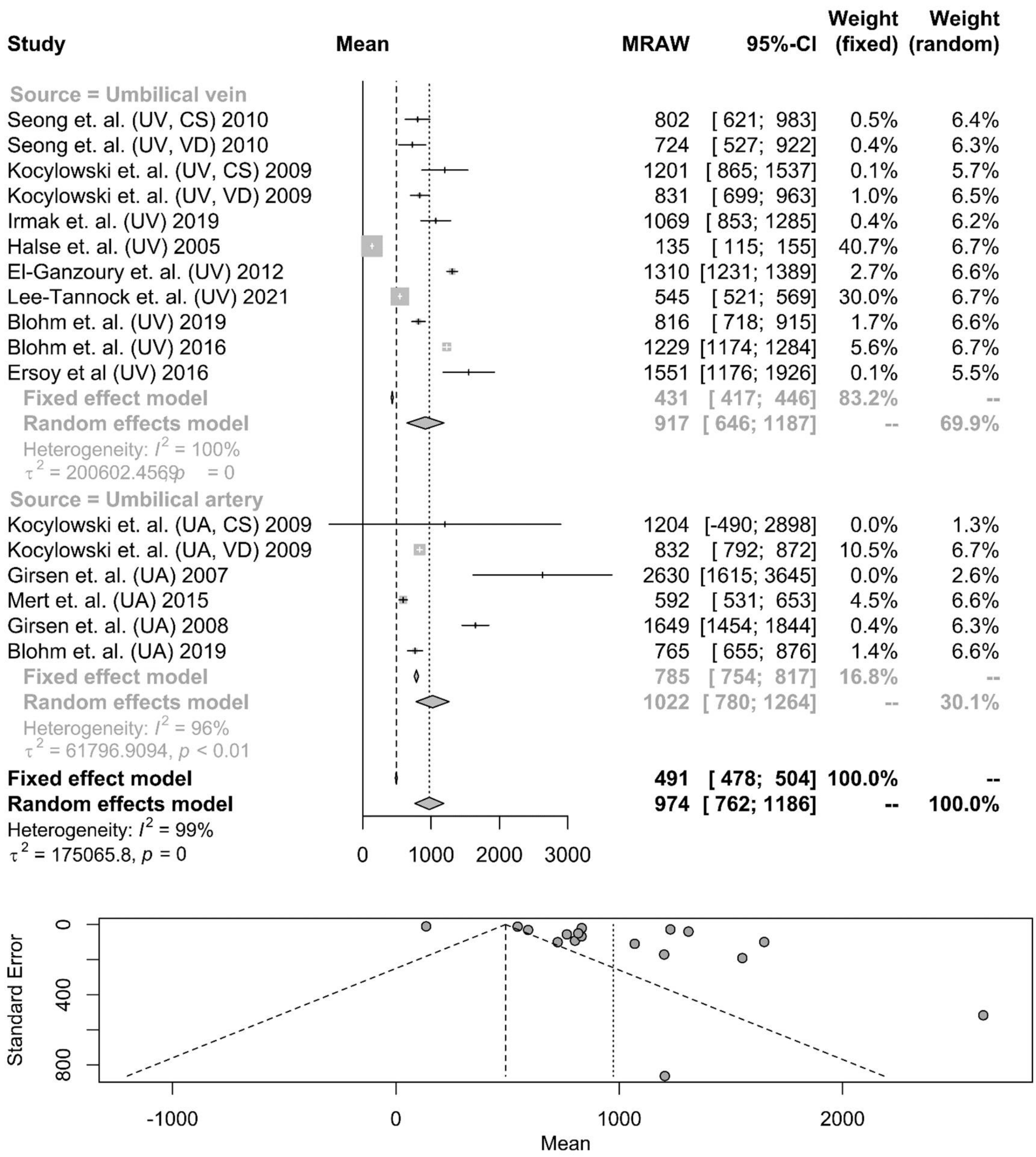

Figure S1: A forest plot that depicts the discrepancy of NT-proBNP concentrations depending on the sampling vessel being the umbilical vein or the umbilical artery, applying the random model or the fixed model. The first column states the first author and the year of publication. The second column represents the mean value and the standard deviation graph. The next three columns show the mean value and the 95%. The remaining two columns present the fixed model and random model weights. The corresponding funnel plot is presented at the bottom.

(UV: Umbilical vein, UA: Umbilical artery, CS: Cesarean section, VD: Vaginal delivery)

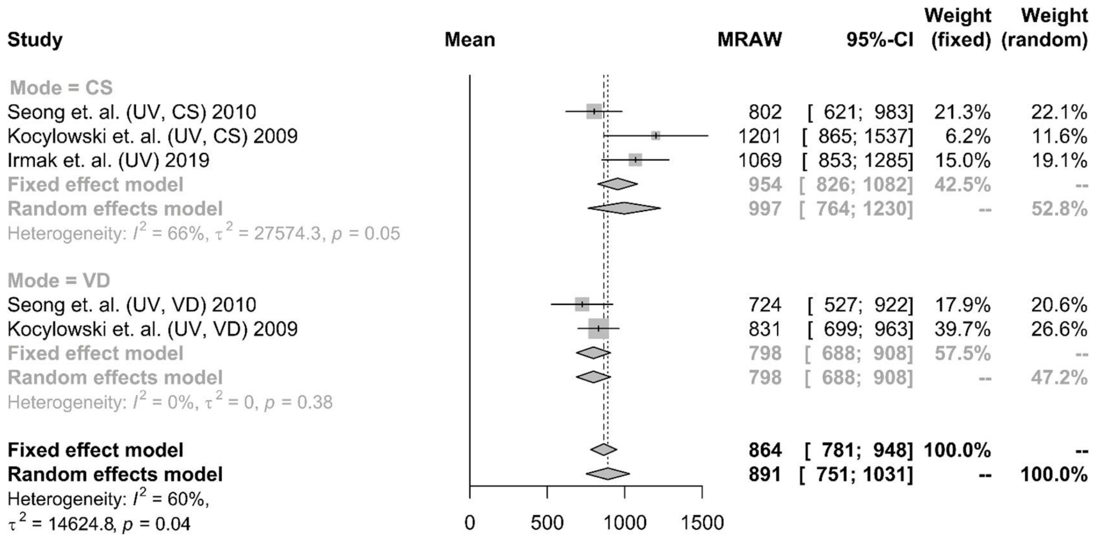

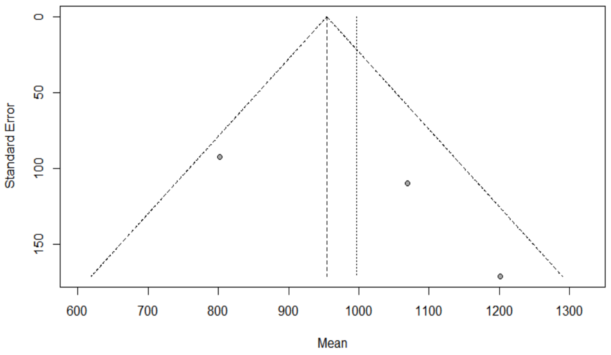

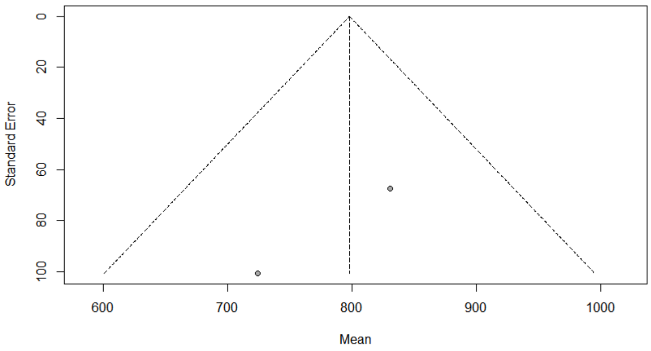

Figure S2: A forest plot that shows the variance of NT-proBNP values according to the mode of delivery, using the random or the fixed model. The first column states the first author and the year of publication. The second column represents the mean value and the standard deviation graph. The next 3 columns show the mean value and the 95%. The remaining 2 columns present the fixed model and random model weights. The corresponding funnel plot is presented at the bottom.

(UV: Umbilical vein, CS: Cesarean section, VD: Vaginal delivery)

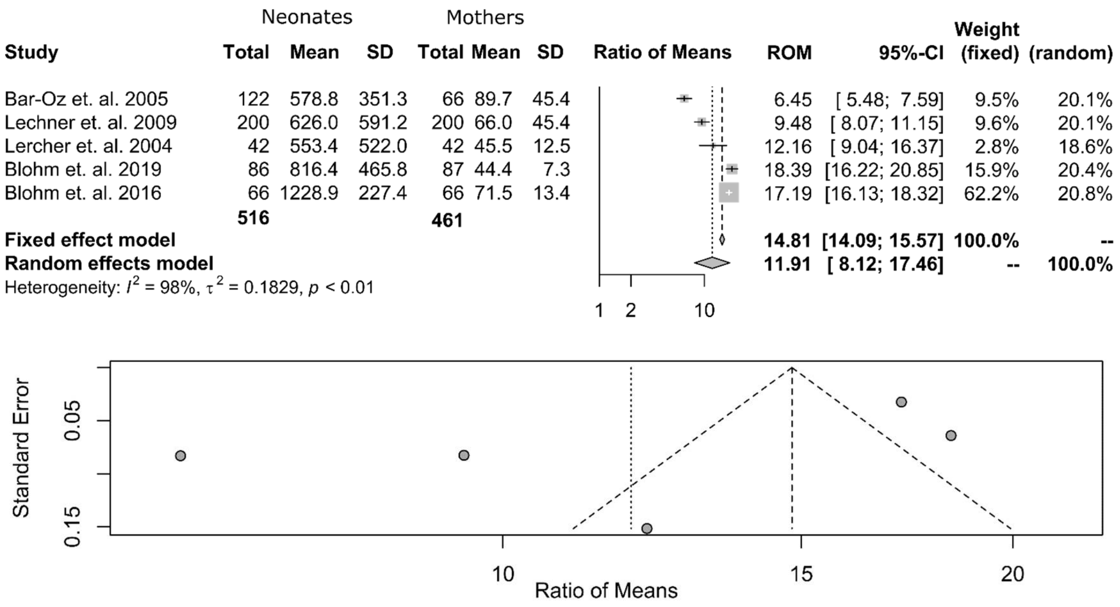

Figure S3: A forest plot that shows the difference between the maternal and umbilical NT-proBNP at birth applying the random model or the fixed model. The corresponding funnel plot is presented at the bottom.
